# Evaluation of polysaccharide intercellular adhesion (PIA) and glycerol teichoic acid (Gly-TA) arisen antibodies to prevention of biofilm formation in *Staphylococcus aureus* and *Staphylococcus epidermidis* strains

**DOI:** 10.1186/s13104-019-4736-8

**Published:** 2019-10-25

**Authors:** Sanaz Amir Gholami, Hamid Reza Goli, Mohammad Reza Haghshenas, Bahman Mirzaei

**Affiliations:** 1 Department of Medical Microbiology and Virology, Faculty of Medicine, Mazandaran University of Medical Science, Sari, Iran; 2 Department of Medical Microbiology and Virology, School of Medicine, Zanjan University of Medical Science, Zanjan, Iran

**Keywords:** PIA/Gly-TA, Mixture, Biofilm formation, *Staphylococcus aureus*, *Staphylococcus epidermidis*

## Abstract

**Objective:**

*Staphylococcus aureus* and *S. epidermidis* as opportunistic pathogens, notable for their frequency and severity of infections are recognized as the most usual reasons for medical device-associated infections that strike hospitalized patients and also immunocompromised individuals. In this study, the polysaccharide intercellular adhesion (PIA) and Glycerol teichoic acid) Gly-TA) as two major macromolecules in the biofilm formation process were purified under the native condition and their structure was analyzed by using colorimetric assays and Fourier Transform Infrared spectroscopy (FTIR). Afterward, the immune response of macromolecules and the mixture of them were assessed by measuring total IgG titers. Subsequently, biofilm inhibitory effects of raising antibodies to biofilm former *S. aureus* and *S. epidermidis* were evaluated.

**Results:**

Obtained data were shown a significant rise in levels of antibodies in immunized mice with mentioned antibodies in comparison with the control group. According to the obtained findings, mentioned antibodies could eliminate *S. aureus* and *S. epidermidis* biofilm formation in vitro assays. This survey confirms the proposal that immunization of mice with a mixture of Gly-TA and PIA vaccine could be secure and protected against *S. epidermidis* and *S. aureus* infection.

## Introduction

Biofilm formation human bacterial pathogens on medical devices, due to the high mortality, morbidity and annually huge costs on the healthcare system are considered as health care concerns worldwide. [1]. Among the biofilm-forming bacteria *S. aureus* and *S. epidermidis*, as etiological agents of 40- 70% of the prosthetic heart valve and catheter biofilm infections, can accumulate in adherent multilayered biofilms, have become resistant to antimicrobial therapy and host defenses [2, 3]. Surface modification approaches including antibiotics, silver, furanones and others, besides, small molecules that can inhibit biofilm formation are documented in the previously published [1]. It had been demonstrated by several experiments that antibodies against staphylococcal cell surface components could be promising to decrease the rate of biofilm formation or adherence of these bacteria to medical devices in vitro [4–7]. Considering Cerca and Litran’s results, immunization of animals with a PIA and conjugate vaccine of PIA gives rise to antibodies that mediated opsonic killing and protected against *S. aureus* and *S. epidermidis* infections [4, 5]. Furthermore, staphylococcal wall teichoic acid especially glycerol teichoic acid (Gly-TA) are known to be associated with serological reactions [8]. Novel preventive solutions (putative biofilm inhibitory candidate antigens) other than the conventional antibiotic therapies and other small molecules to eradicating medical devices related infection (MDRI) are urgent to prophylaxis of mentioned structure especially after the entrance of bacterial agent in high-risk patient’s body [1]. Then, the potential of mouse polyclonal antibodies rose against the PIA, Gly-TA and the mixture of PIA and Gly-TA for the eradication of *S. aureus* and *S. epidermidis* biofilms in vitro got measured.

## Main text

### Materials and methods

#### Extraction and purification of PIA and Gly-TA

Extraction and purification of PIA and Gly-TA were accomplished basing on the previously published [9]. In short, growth *S. epidermidis* strains colonies in 2 L of trypticase soy broth (TSB) (37 °C for 24 h under moderate shaking (40–50 rpm/min) were harvested by centrifugation (4500 rpm, 20 min, 4 °C), and cells resuspended in 20 ml of PBS (pH 7.5). Then the preparation was sonicated four times for 30 s on ice. After centrifugation (12,000 rpm, 15 min, 4 °C) the supernatant was dialyzed (12 KD) overnight against the same buffer and was concentrated by the use of Centriprep 10 (Amicon, Witten, Germany). Following the elimination of soluble proteins by using proteinase-K, sample was directly loaded onto an equilibrated 1.6-by 100-cm Sephacryl S-100 (Pharmacia LKB GmbH, Freiburg, Germany) with 50 mM sodium phosphate. Finally, purified macromolecules were stored at − 20 °C [9]. Contaminating DNA, RNA, and protein in purified PIA and Gly-TA were eliminated by enzymatic digestion.

#### Confirmation of purified PIA and Gly-TA by Colorimetric assay

By this procedure the hexosamine present in glycosaminoglycan’s under conditions of mild acid treatment (pH = 5) was analyzed. The interaction between 3-methyl-2-benzothiazolone hydrazone hydrochloride (MBTH) and the 2,5-anhydrohexoses produced by the deamination of hexosamines was evaluated based on the formed color complex [10]. Furthermore, the carbohydrate content of Gly-TA was determined by phenol sulphuric acid using a standard curve of glucose [11].

#### Confirmation representative Gly-TA and PIA by FTIR

Infrared spectroscopy of purified polysaccharides was investigated using the regularized method of deconvolution. Briefly, powdered samples were dispersed in KBr pellets and recorded with a TENSOR 27 Bruker instrument, averaging of 256 scans on the FTIR spectrometer [12].

#### Pyrogenicity test, Toxicity, and general safety

Pyrogenicity [13] and toxicity [14] of antigens was accomplished based on the previous publishes. The amount of endotoxin in the prepared antigens was measured by a commercial Limulus amebocyte lysate kit (Thermo Scientific, Waltham, MA, USA) according to the manufacturer’s recommendations as well [14].

#### Immunization of mice

Female BALB/c inbred mice, 6–8 weeks old were divided into four groups consisting of 6 mice. The mice supplied with a standard diet and water. Each mouse in the specific group was immunized three times subcutaneously on days 0, 14 and 28 with 100 µg (by using serial dilution procedure and evaluation by colorimetric assays) of the respective lyophilized antigens in 1% alum dissolved in PBS (filtered at 0.22 nm pore diameter). Boosters were injected 2 and 4 weeks after the later first immunization. Two weeks after each injection, (500 µL) blood was obtained from orbital sinus from the six mice in each group. Each time, to get serum the blood was centrifuged at 5000 rpm for 5 min and was stored at − 20 °C [15]. Each antigen was also passed through a 0.22 micron filter before immunization of mice.

The experimental groups were as follows:

- G-I: PIA (100 µg);
- G-II: Gly-TA (100 µg);
- G-III: PIA/Gly-TA mixture (50/50 µg);
- G-V: PBS.

#### Enzyme-linked immunosorbent assay (ELISA)

Persistence of anti-PIA and anti-Gly-TA antibodies in the immunized mice sera was achieved by applying commercial enzyme-linked immunosorbent assay (ELISA) kit (Sino Biologic, Inc,) conforming to the manufacturer instructions by coating the 1 μg/well of PIA and an appropriate dilution of capture goat anti-mouse IgG antibody overnight at 4 °C. Then the procedure was done based on the previous published [16]. The absorbance at 492 nm was measured and the amount of antibodies was predicted by comparing the control group based on the optical density. Each test diluted sera was repeated three times in the 1:2–1:1024 serial dilution.

#### In vitro biofilm inhibition assay

Taking advantage of a semi-quantitative microtiter plate method, the effect of pre- and post- immune IgGs against injected antigens on in vitro biofilm formation to biofilm-forming *S. epidermidis* 1457 and an *S. aureus* biofilm-forming wild type strain was studied [6]. Sterile TSBg, as a negative control, was included and the assay and was independently repeated three times [7, 17]. In vitro biofilm formation assay previously described by published as well [6].

By the use of the following formula the percent inhibition of biofilm formation was calculated [15]:

$${{\left( {{\text{A 595}},{\text{ positive }} - {\text{A 595}},{\text{ antibody}}} \right)} \mathord{\left/ {\vphantom {{\left( {{\text{A 595}},{\text{ positive }} - {\text{A 595}},{\text{ antibody}}} \right)} {\left( {{\text{A 595}},{\text{ positive }} - {\text{A 595}},{\text{ negative}}} \right)}}} \right. \kern-0pt} {\left( {{\text{A 595}},{\text{ positive }} - {\text{A 595}},{\text{ negative}}} \right)}} \times 100.$$

#### Statistical analysis

Utilizing multiple- group analysis of variance (ANOVA), statistical analysis of results by using Graph pad prism was accomplished and a *P* value < 0.05 was considered significant.

#### Euthanasia method

Using the halogenated ether procedure as an inhalant anesthetic, mice were euthanized as well. In short, mice were Anastasia by halogenated ether and then euthanized by encountering to inhalant ether overdose (up to 2–5% to effect).

### Results

#### Purified PIA and Gly-TA properties

PIA and Gly-TA were purified at a flow rate of 1 ml/min and fractions of 5 ml were collected over 5 min in a fraction collector. (Additional file 1). Purified PIA underwent chemical analysis, it was indicated that it contained 65% hexosamine (5700 µg/ml), this amount to Gly-TA was 2300 µg/ml based on the phenol sulphuric colorimetric assay. Using FTIR The composition and structure of PIA and Gly-TA were confirmed. (Additional files 2, 3, 4).

#### Endotoxin contents and pyrogenicity and toxicity test

There were no overt signs of toxicity or pyrogenicity after i.p. or intravenous administration (separately) of the inoculum to animals. The content of endotoxin based on the Limulus amebocyte lysate test was determined as 4.5 EU/mL.

#### Anti-antigens humoral response

**Fig. 1:**
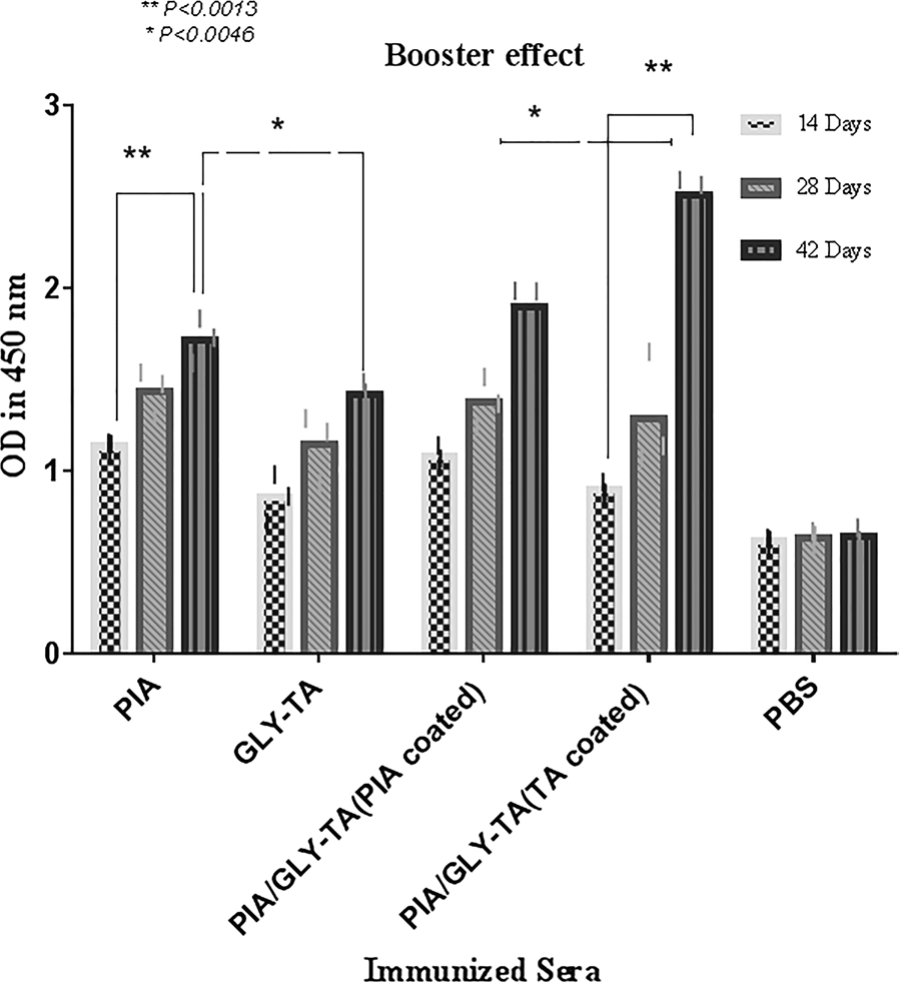
The booster effect of the assessed antigens at different times. ELISA was performed by coating the native PIA and Gly-TA and the increase of the antibodies was assessed for immunized sera compared to the controls. Significant effects were observed from use of the mixture and conjugate booster. Six weeks after the first injection, the titres of antibodies had increased. The error bar is representative of the mean ± SD (n = 3)

Using antigen mediated ELISA in order to evaluate the total IgG antibody response against PIA in the antigen mixture, mice sera antibodies titers were determined (≤ 1:200). Following the first immunization by Gly-TA (*P *= 0.0567), PIA (*P *= 0.0138) and mixture (*P *= 0.2142), low-level IgGs production comparing to the control group was observed and this amount determined as not significant to Gly-TA and mixture (PIA/Gly-TA) immunized sera. An increase in IgG titres to PIA (*P *= *0.*0021), Gly-TA (*P *= *0.0017*) and mixture (*P *= *0.0123*) was observed following the first booster. The third group of mice received a mixture of PIA and Gly-TA and an increase in anti-PIA antibodies was observed following the second immunization (*P *< 0.0003). The difference of arisen antibodies between the immunized sera was specified in Fig. 1.

#### In vitro biofilm inhibition assay

**Fig. 2:**
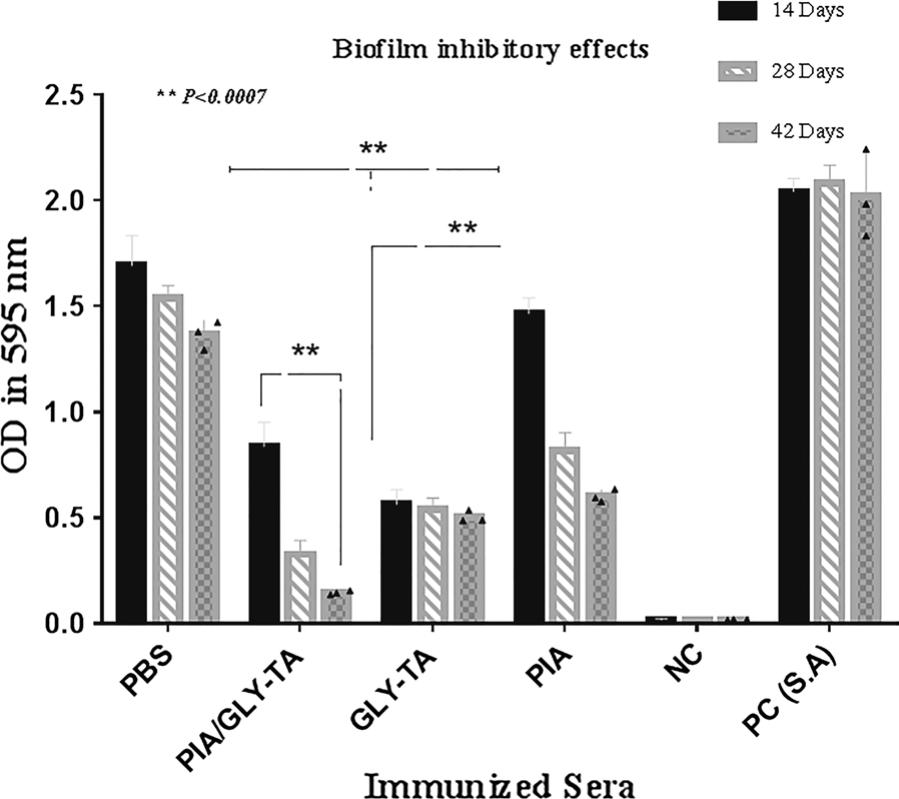
Comparative analysis of biofilm inhibitory effects to a biofilm forming *S. aureus* wild type of the diluted sera. The biofilm-inhibitory effects of increasing antibodies (after each shot) were determined by comparing the positive and negative controls using the mentioned formula. The error bar is representative of the mean ± SD (n = 3). Biofilm inhibition was induced by the antibodies (14/28 days) in the PIA (*P *< 0.0004), Gly-TA (*P *= 0.0002) and mixture of them (*P *> 0.0024) were statistically significant as well

**Fig. 3:**
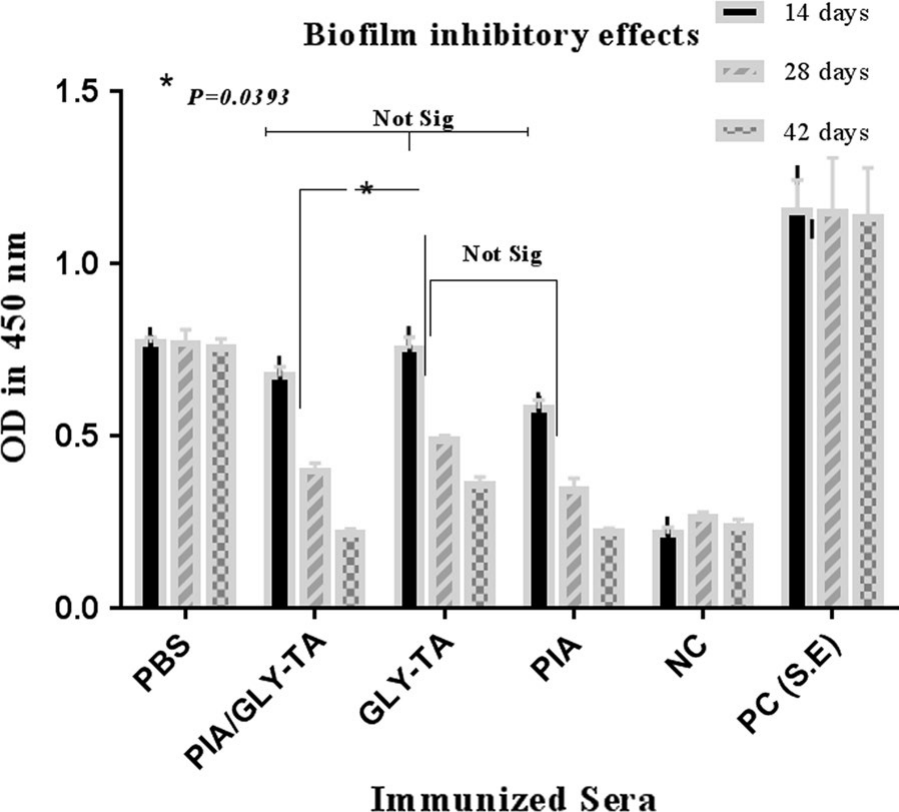
Biofilm inhibitory effect of immunized sera to a biofilm former *S. epidermidis* (1457)

Pre and post-immune sera were tested in order to evaluate the biofilm inhibitory effect by semi-quantitative biofilm inhibition procedure. Experiments were observed with sera from mice that were boosted after specific time spans (14, 28 and 42 days) and results of immunized and non-immunized sera were compared. Data showed that the sera of the mice immunized with the mixture of PIA and Gly-TA gave significant inhibition (*P *< 0.0002) after the second booster. The effect after the first injection of the mixture was also significant (*P *> 0.0111). Inhibitory effects of sera from mice injected with PIA (*P *> 0.0001), Gly-TA (*P *> 0.0001) were also significant after the second booster. In vitro biofilm inhibition assay showed that there is no distinction between the immunized groups and these differences were not considered. Detailed data to biofilm inhibitory effect and differences of its, were indicated in Figs. 2, 3.

### Discussion

In this study, we tried to identify the new potential target(s) for immunoprophylaxis and immunotherapy against biofilm-forming *S. aureus* and *S. epidermidis*. The production of the PIA and Gly- TA has been directly associated with the accumulation phase of the bacteria population in the biofilm formation procedure. In our survey, two PIA positive and negative strains were grown as parallel in the same conditions and the targeted polysaccharide was purified. Our results confirm previously published findings [5, 8]. PIA and Gly-TA were purified at the flow rate of 1 ml/min and fractions of 5 ml were collected in 5 min with a fraction collector. PNAGII or PIA roughly has 100KD and is soluble in buffers according to Litran et al. findings [18]. Given the effect of removing impurities from the antigen to increasing functional antibodies in this study, the impurities were done with additional centrifugation [5, 19]. Considering FTIR patterns, regions where stretching vibrations ν(CO) C–O–C glycosidic bridge in oligosaccharides manifest are present in the spectra of the 1175-1140 cm-1 ranges, This range is close to the previously mentioned in work [20]. In the spectra’s, the C=O stretch band has been demonstrated in 1739 (PIA) and 1651(Gly-TA)/cm data demonstrated.

In spite of PIA being immunogenicity poor, biofilm inhibitory effects have been reported from raised antibodies to this antigen. In this study according to acetylation of polysaccharide (PIA) and the presence of amine groups, the titer of IgGs were enhanced after boosters, but these IgGs did not show effector activity compared mixture, since a significant rise in the IgG titers in the PIA/Gly-TA (mixture) vaccinated group against PIA was expected. The 14th day sera did not show biofilm inhibitory effects against *S. epidermidis* biofilm formation, these effects statistically were significant in *S. aureus* (*P *< 0.0402), while after receiving the boosters a significant inhibition was observed in tested sera by S. aureus compared to 14 days immunized sera. Mentioned Antigens raised antibodies and protection and also enhanced the levels of protective IgG titers. Since one of the most important characteristics of vaccine candidates is the in vitro and in vivo effects of them, in this study, the features of polyclonal secreted antibodies analyzed on biofilm formation under laboratory conditions. Data showed that immune sera after 14 days had *S. aureus* biofilm inhibitory effect, this amount was not statistically significant (*Ps *> 0.257) to *S. epidermidis* when compared to other immunized sera groups. Based on the Litran et al. [5] findings, PNAGII couldn’t raise the antibodies but in our result however at the first immunization comparing the control although, rising the antibodies was not statistically significant but at the first and second booster dosage amount of arisen antibodies were increased. Removing impurity in PIA by additional centrifugation and exposing the amine functional group of PIA could affect this phenomenon [5].

Polysaccharides antigens in some bacteria arouse low immune responses and vaccination by polysaccharide needs to be boosted, mainly in the elderly and in infants. Although the antigenicity of most polysaccharides is poor, increased antibodies to PIA have shown a biofilm inhibitory effect [13]. In this study, the functions of elicited antibodies were assessed by the in vitro biofilm inhibition protocol. It was observed that the inhibitory effect of the mixture statistically was significant (*P *< 0.0024). However, after the first and second booster, the effect of biofilm inhibition in the mixture group and other groups showed a significant difference compared with the control group in the last booster. Efficacy of antibodies in immunized sera showed that, at the first immunization for PIA (*P *= 0.0004), the biofilm inhibitory effect was statistically significant when compared with the control group. Reports indicated that the mixture of mentioned macromolecules had a good biofilm inhibitory effect.

## Limitations

At the current study challenging of arisen antibodies to in vivo challenge and isotyping of the IgGs due to financial and time constraints were not accomplished. It seems that more research is needed for the determination of antibodies isotypes and challenging experiments.

## Supplementary information

**Additional file 1.** Purification and analysis of PIA and Gly-TA. Representative fast protein liquid chromatography (FPLC) chromatogram for native PIA and Gly-TA. PIA was eluted 78 min after injection time. Gly-TA, 42 min after suspected PIA peak was eluted. Polysaccharide and protein were identified at the 206 and 280 nm wavelengths.

**Additional file 2.** IR spectra of PIA macromolecule in the 4000–500/cm range and the result of this deconvolution. According to the composition of the PIA molecules presence of C=O groups of the native polysaccharide and determination of N–H amide bond and PIA anomeric carbon in the FTIR pattern, the composition of the PIA was confirmed.

**Additional file 3.** The composition of Gly-TA in IR spectra in the 4000–500/cm range and the result of this deconvolution.

**Additional file 4.** IR spectra of PIA/Gly-TA mixture in the 4000–500/cm range and the result of this deconvolution.

## Data Availability

All the results of this study have been classified and maintained by the dissertation in the Mazandaran University of Medical Sciences. We have indeed provided all raw data on which our study is based. Competing Interests: The authors declare that they have no competing interests.
